# Barriers to utilisation of antenatal care services in South Sudan: a qualitative study in Rumbek North County

**DOI:** 10.1186/s12978-017-0327-0

**Published:** 2017-05-22

**Authors:** Calistus Wilunda, Chiara Scanagatta, Giovanni Putoto, Francesca Montalbetti, Giulia Segafredo, Risa Takahashi, Serge André Mizerero, Ana Pilar Betrán

**Affiliations:** 1 Department of Pharmacoepidemiology, Graduate School of Medicine and Public Health, Kyoto University, Yoshida Konoe-cho, Sakyo-ku, Kyoto, 606-8501 Japan; 2 Doctors with Africa CUAMM, Via San Francesco 126, 35121 Padova, Italy; 3 Doctors with Africa CUAMM, c/o DHL Worldwide express, Abdul Hakam Tayfour Building, Juba, South Sudan; 4 Department of Nursing Science, Faculty of Health Care, Tenri Health Care University, 80-1 Bessho-cho, Tenri City, Nara, 632-0018 Japan; 5 Department of Global Health and Socio-epidemiology, Graduate School of Medicine and Public Health, Kyoto University, Yoshida Konoe-cho, Sakyo-ku, Kyoto, 606-8501 Japan; 6 UNDP/UNFPA/UNICEF/WHO/World Bank Special Programme of Research, Development and Research Training in Human Reproduction, Department of Reproductive Health and Research, World Health Organization, Avenue Appia 20, 1211 Geneva 27, Switzerland

**Keywords:** Maternal health, Reproductive health, Pregnancy, Fragile states, Post-conflict settings

## Abstract

**Background:**

Access to adequate antenatal care (ANC) is critical in ensuring a good maternal health and in preventing maternal and neonatal morbidity and mortality. South Sudan has one of the world’s poorest health indicators due to a fragile health system and a combination of socio-cultural, economic, and political factors. This study was conducted to identify barriers to utilisation of ANC services in Rumbek North County.

**Methods:**

Using a qualitative design, data were collected through 14 focus group discussions with 169 women and 45 men and 12 key informant interviews with community leaders, staff working in health facilities, and the staff of the County Health Department. Data were analysed using inductive content analysis.

**Results:**

The perceived barriers to ANC utilisation were categorised as follows: 1) Issues related to access to health facilities and lack of resources. These included long distance to health facilities, lack of means of transportation to the health facilities, floods and poor roads, and demand for payment for health care at some health facilities; 2) The influence of the socio-cultural context and conflict including heavy burden of domestic chores, the negative influence of husbands who were reluctant to allow their wives to attend ANC, and insecurity; 3) Perceptions about pregnancy including misperceptions about the benefits of ANC and low perceived risk of pregnancy-related complications; and 4) Perceptions about the quality of care and the efficacy of medical treatment.

**Conclusions:**

This study identified a myriad of factors deeply entrenched in the society, which prevent women from utilising ANC services. It also elicits broad aspects of interconnectedness among the barriers. To ensure effectiveness, strategies to improve utilisation of ANC in the study area and in similar contexts need to take into account the barriers identified by this study.

**Electronic supplementary material:**

The online version of this article (doi:10.1186/s12978-017-0327-0) contains supplementary material, which is available to authorized users.

## Plain English summary

This study was conducted to identify the reasons why pregnant women in Rumbek North do not attend clinics for health check-ups and care during pregnancy. Data were collected through 14 group discussions with women and men separately and 12 one-on-one interviews with community leaders, staff working in health facilities, and the staff of the Rumbek North County Health Department. The reasons why pregnant women were not attending clinics included long distance to health facilities, lack of transport means, flooding and poor roads, demand for payment at health facilities, heavy burden of domestic work among women, the negative influence of husbands who were reluctant to allow their wives to attend ANC over such concerns as the safety of the woman and of the children left at home, insecurity, feelings about the benefit of care during pregnancy and of the danger of health problems during pregnancy, and the views about the quality of care and the benefit of medical treatment. In order to succeed, strategies to improve utilisation of clinics for care during pregnancy in Rumbek North should address the issues identified in this study.

## Background

Maternal health remains one of the unmet challenges on the global development agenda [1]. Access to adequate antenatal care (ANC) is critical in ensuring a good maternal health and in preventing neonatal morbidity and mortality [2]. The World Health Organization (WHO) currently recommends a minimum of eight ANC visits instead of the previously recommended minimum four visits [3]. South Sudan, the world’s youngest nation, is plagued by a myriad of challenges including a fragile health system, which has been aggravated by decades of armed conflict, resulting in a poor health status of the population. According to the 2010 Sudan Household Survey (SHHS), only 40.3% of pregnant women received any ANC by a skilled provider [4]. The determinants of ANC services utilisation in the country are still poorly understood. So far, only one published study, based on data from the 2010 SHHS, has attempted to explore the factors associated with non-use of ANC [5]. More information is needed for evidence-based planning of ANC service delivery in this context.

The Government of South Sudan has launched various policies and strategies to address the health system challenges faced by the new nation. The Health Sector Development Plan (HSDP) 2012–2016 aimed to “contribute to the reduction of maternal and infant mortality and improve the overall health status and quality of life of the South Sudanese population” [6]. One of the objectives of the HSDP was to increase the utilisation and quality of health services with an emphasis on maternal and child health. To operationalise the HSDP, the government defined a Basic Package of Health and Nutrition Services (BPHNS), which contains a set of interventions aimed to reduce morbidity and mortality in South Sudan [7]. These interventions are grouped into four categories. The first one is “Integrated Reproductive Health Care”, which includes essential obstetric care as one of its components. The BPHNS further lists the services to be provided at each level of the health system together with the required human and material resources. Although South Sudan has subsequently released a number of health policy documents, the BPHNS remains the basic framework for health service provision in the country. Despite the government’s efforts, the health system in South Sudan is still faced with many problems such as lack of human resources, poor infrastructure, shortage of drugs and supplies, and weak management [8]. To mitigate some of these challenges, the government works with non-governmental organisations, based on the “contracting out” model [9], to provide primary health services in the country [6]. From 2013, the Ministry of Health (MoH) in Rumbek North County started to partner with Doctors with Africa CUAMM (hereafter CUAMM) to implement a comprehensive primary health care project. The project began by reactivating health facilities which had been closed or were partially functioning due to lack of staff, equipment, and supplies. The reactivation involved infrastructural renovations; staff recruitment, on-the-job training and supervision; and provision of equipment, drugs and supplies.

Routine data collected by the MoH in the county showed that, in 2014, 54.8% of pregnant women received any ANC, 11.3% attended four or more ANC visits and 1.7% delivered in a health facility. To design a strategy to improve maternal health services delivery in the county, we conducted a study to understand the barriers to utilisation maternal health services (ANC and childbirth). The findings on the barriers to utilisation of childbirth services have been reported elsewhere [10]. In this paper, we report the findings of a qualitative study designed to elicit the barriers to utilisation of ANC services.

## Methods

This study is reported per the consolidated criteria for reporting qualitative research (COREQ) [11]. The detailed methodology (including the COREQ checklist) has been described elsewhere [10]; below is a summary.

### Study area

This study was conducted in Rumbek North County, which in 2015 had a population of 59,740 inhabitants [12], and was divided into six *payams* (sub-county units): Alor, Malueth, Mayen, Madol, Maper and Wunrieng. The county’s population is semi-nomadic and pastoralism is the main economic activity. In 2015, the county had one Primary Health Care Centre (PHCC) located in Maper, and seven Primary Health Care Units (PHCUs). Each one of the PHCUs was run by one community health worker (CHW), one traditional birth attendant (TBA), and one drug dispenser. The PHCC had three expatriate professional health workers: a nurse, a midwife, and a clinical officer. In South Sudan, PHCUs and PHCCs are, respectively, the lowest- and second lowest-level health facilities of the health system. These health facilities are mandated to provide ANC services [7]. Each PHCU is supposed to be staffed by two CHWs and a community midwife while a PHCC is supposed to have one clinical officer, three professional nurses, two midwives, three CHWs, and lower cadre staff [7]. At the time of this study, a complete package of ANC services, apart from laboratory tests, was being provided daily at the PHCC and at four PHCUs and through scheduled outreaches at the rest of the PHCUs. In South Sudan, the ANC package at PHCUs and PHCCs includes identification of pregnant women and rising awareness on early initiation and compliance with ANC, provision of services for prevention of mother to child transmission of HIV, prevention and treatment of sexually transmitted infections, nutrition counselling and micronutrient supplementation, malaria prevention interventions, identification and referral of women at high risk, and monthly outreach clinics [7].

### Design, sampling, and participants

This qualitative study collected data through focus group discussions (FGDs) in eight randomly selected villages and through key informant interviews (KIIs). Villages in the county were stratified by *payam* and randomly selected as follows: two villages from each of Malueth and Mayen *payams* (the most populous) and one from each of Madol, Alor, Maper and Wunrieng. In each village, one FGD was conducted with women aged 18 years and above who had delivered in the preceding 12 months and were usual residents in the county. Additionally, in a random sub-sample of half of the villages, husbands of women who delivered in the preceding 12 months were recruited to participate in men’s FGDs. Two extra FGDs with women were conducted in one cattle camp. In each selected village, CHWs invited 12 eligible participants to take part in the FGDs and all those who turned up were included. KIIs were conducted with a purposive sample of CHWs in the PHCC and PHCUs, community leaders, and staff of the County Health Department (CHD). The choice of the number and type of participants to be enrolled in this study depended on the extent to which they could contribute to providing relevant information in response to the research questions [13].

### Data collection

FGDs and KIIs were conducted in March 2015 utilising open-ended pretested question guides. Each FGD was conducted by two Dinka-speaking facilitators who were previously unknown to participants. The facilitators were of the same gender as participants, had at least high school-level education, and were conversant with the local language and culture. One data collector facilitated the sessions while the other one managed audio recordings and took field notes. The data collectors were trained for 1 day and were supervised by one of the co-authors (CW) who is experienced in qualitative research. The FGDs were held in local church structures and under tree shades. KIIs took place at venues that were convenient to participants.

A total of 14 FGDs with 45 men (4 FGDs), 127 women in the villages (8 FGDs), and 42 women in cattle camps (2 FGDs) were conducted. The characteristics of FGD participants have been described previously [10]. In brief, the women FGD participants had a median age of 25 years. A majority had no education (96.7%); were married (92.6%); and had attended at least one ANC visit during their most recent pregnancy (67.5%). The median age of male FGD participants was 35 years and 71% of them had no formal education. Twelve KIIs were conducted with the following individuals: 3 community leaders, 3 PHCU staff, 4 PHCC staff, and 2 CHD staff. Women’s FGDs had a median of 16 participants while men’s FGDs had a median of 9 participants. There were no drop-outs during FGDs. All KIIs were conducted by one of the co-authors (CW) either directly in English (for CHWs and CHD staff) or through a translator (for community leaders). Both KIIs and FGDs were audio recorded. Each FGD session lasted for about one hour whilst each KII lasted for about 20 min. No repeat interviews were conducted.

### Data analysis

Audio recordings in Dinka language were transcribed and translated into English by bilingual (Dinka and English) speakers while audio recordings of KIIs conducted in English were transcribed by CW. The transcripts were not returned to participants for review because of logistical constraints. The transcripts were then analysed using the inductive content analysis approach [14]. The analytic framework was adapted from a large systematic review [15]. Although the original framework is about barriers to childbirth service use, the themes were modified to apply to ANC use. Coding was done using NVivo 10 (QSR International, Melbourne, Australia). Information from KIIs was used to triangulate findings from FGDs. The data for each theme and sub-theme were then pieced together to provide an overview of the content relating to that specific theme (charting). The four broad themes were: 1) access and resource availability, 2) influence of the sociocultural context and insecurity, 3) Perceptions of pregnancy, and 4) perceptions of the quality of care. Quotes were selected to represent a typical response or to illustrate a deviant opinion.

## Results

Table 1 summarises the findings of the study. The perceived barriers to utilisation of ANC services in the county are described in detail below. FGD participants often used the word “hospital” to refer to any type of health facility.

**Table 1:** Barriers to utilisation of antenatal care in Rumbek North County, South Sudan

Barrier	Main findings
a. Access and resource availability	
1. Transportation/access	
1.1 Proximity of health facility	Long distance to heath facilities aggravated by sparsely distributed population settlements. A semi-nomadic lifestyle which increased the distance to health facilities. Some nearby PHCUs not providing ANC.
1.2 Transport means availability	Lack of commercial or private means of transportation.
1.3 Flooding and poor roads	Floods and mud during the wet season, inability to swim, parts of roads being washed away, inaccessibility of health facilities for delivery of drugs and supplies, bumpy roads which predisposed women to excessive shaking if travelling by car or motorbike.
2. Costs	Pregnant women being asked to pay money at some health facilities. Having been asked to pay for ANC in the past affecting current ANC service use. Transportation costs to health facilities.
b. Influence of sociocultural context and conflict	
1. Domestic chores of women	Lack of time to attend ANC due to the heavy burden of domestic work. Lack of someone to leave behind with children if a woman decides to visit a heath facility. Inability to arrive at the health facility on time due to domestic chores.
2. Influence of husbands/male partners	Men unwilling to pay for costs associated with visiting a health facility, men restricting their partners from attending ANC, lack of emotional support and encouragement from men, lack of interest in maternal health by men. Men perceive ANC attendance to be unnecessary because foremothers never used to attend ANC.
3. Insecurity	Frequent attacks by neighbouring tribes/clans. Constant fear of being attacked at any time by neighbouring tribe/clans. Women cannot leave children at home alone to attend ANC because of the insecurity. Husbands cannot allow their wives to attend ANC because of insecurity. Displacement after attacks exacerbated geographic inaccessibility.
c. Perceptions of pregnancy	
1. Perceived benefit	ANC perceived to be a new concept in the community, unfamiliarity with ANC and its significance, lack of prior contact with the formal heath system.
2. Perceived risk	Low-risk perception due to no prior pregnancy-related complications and trivialization of health problems during pregnancy. ANC attendance not viewed as a routine exercise but linked to pregnancy complications.
d. Perceived quality of care and efficacy of medical treatment	Dissatisfaction with ANC if medical treatment was not provided during the visit. Attendance of ANC influenced by whether the woman’s symptoms were relieved by treatment received during previous ANC visit.

A. Access and resource availability
  Transportation/access
    Transportation/access issues included long distance to health facilities, flooding and poor roads, and lack of transportation means. Long distance to health facilities was occasioned by the sparseness of the population settlements, nomadism in search for water and pasture, and the lack of enough facilities providing ANC in the county.
*“Another reason why women don’t attend ANC is the long distance from villages to health facilities. Some villages and payams have no health facilities and women are discouraged from visiting faraway health facilities.”* (Male FGD participant, Maper centre)
*“Most of the ladies stay in cattle camps and are used to migrating every season to other areas. In the dry season, they have to go to a far place where there is water and during the rainy season, they move to a place where there are no mosquitoes because this place has a lot of mosquitoes if there is water.”* (KII, CHD staff)
    Long distance and lack of means of transportation to health facilities aggravated the effect of the other barriers on ANC attendance (mentioned below). Women felt that if a health facility was near, they would attend ANC and return home quickly without having to worry about insecurity. Additionally, their husbands would be less likely to restrict them from attending ANC.
*“It is mainly a matter of distance. If you bring a hospital near, our problems will be solved. We will not be worried about our children, husbands, and cattle.”* (Female FGD participant, Chatom village)
*“Most of the time, we don’t have transportation means to the hospital in Maper or Rumbek.”* (Female FGD participant, Wundhiot village)
    Flooding during wet seasons prevented women from accessing health facilities as highlighted below:
*“During the wet season, the land becomes flooded and the water can reach the shoulder level. If you are pregnant, you cannot swim in such a place, because your heart will get tired and your thighs will be exhausted.”* (Female FGD participant, Chatom Village)
    Additionally, flooding prevented delivery of drugs and supplies to health facilities rendering them functionless. Floods washed away parts of the main road connecting Maper centre and Rumbek town causing serious logistical challenges.
*“I did not go to the hospital because I was in the village and the land was flooded with water and there were no drugs that are usually given to pregnant women at the health facility.”* (Female FGD participant, Meen village)
    There were also concerns about the excessive shaking that occurs when travelling by a motor vehicle on the rutted roads in the county.
*“I did not go to the hospital in the past month because the roads were very bad. You can neither walk on foot nor use the car because they say that pregnant women are not supposed to get in the car if there is too much shaking due to bad roads.”* (Female FGD participant, Meen Village)
  Costs
    Both male and female FGD participants mentioned that women were discouraged from attending ANC because of being asked by health facility staff to pay money. It was unclear whether these payments were official or under-the-table payments. Participants, however, noted that this practice was not present at all health facilities, and was being encountered mainly at health facilities located in neighbouring counties.
*“Some health facilities ask pregnant women to pay fees. This can discourage women from attending antenatal care because they don’t have money.”* (Male FGD participant, Maper centre)
*“I tried going to the health facility and I was told by the man I found there to bring money.”* (Female FGD participant, Madhol village).
    The experience of having been asked to pay for ANC or for treatment during pregnancy was negatively affecting the current use of ANC services.
*“In the past when people were being asked to pay money, we did not use to go to the hospital because we did not have money to carry with us. We did not even have one pound to buy soap, leave alone money to pay at the hospital. If a pregnant woman is asked to pay money every day she goes to the hospital, but she is very poor and cannot afford to pay, do you think she will go again?”* (Female FGD participant, Maper centre)
    Some women who had been asked to pay for services returned home and spread this message to their friends and relatives; discouraging more women from attending ANC.
*“My sister went to the hospital and when she came back she told me that I should never go there if I did not have money.”* (Female FGD participant, Madhol village)
B. Influence of the sociocultural context and insecurity
  Domestic chores of women
    Women in Rumbek North bear a heavy burden of domestic chores. They were responsible for taking care of children, taking care of the house, and producing and preparing food for the family. Domestic chores increased substantially during planting, weeding and harvesting seasons when women were required to work on their farms besides attending to their families. A list of the duties of women and men in Rumbek North is presented in Additional file 1. Long distance to health facilities and insecurity worsened the problem of domestic chores. There was also the problem of lack of someone to take care of children at home if a woman wished to attend ANC.
*“Sometimes we are preoccupied with work at home because we are the ones who are responsible for all the work and we have no time to visit the health facility, unless if it is near. The other reason is that there is nobody to leave our children at home with.”* (Female FGD participant, Achiek village)
    Because of domestic chores, some women could not arrive at health facilities during the regular time of service delivery and there were concerns about being turned away for arriving too late at health facilities.
*“Children cannot spend the whole day without eating. So you spend time cooking in the morning until the hospital visiting time is over. If you go to the hospital late, doctors will say that the time for registration is over. Therefore, women who work in the morning may not go to the hospital because they can only arrive there late every day.”* (Female FGD participant, Maper centre)
  Influence of male partners
    Partners of pregnant women had a great influence on utilisation of ANC and other maternal health services in Rumbek North. Because men controlled the resources of the family, they were often unwilling to pay for the costs associated with health facility visits. Both male and female FGD participants mentioned that men were restricting their wives from attending ANC based on three main factors: 1) long distance to health facilities: men did not want their wives to travel to faraway health facilities leaving their homes and children unattended, 2) men did not want their wives to travel to health facilities because of insecurity, and 3) some men did not see the necessity of their wives attending ANC because their mothers never used to do so.
*“Our husbands do not know the benefits of the hospital. They keep us at home saying that ‘our mothers never used to go to the hospital and were giving birth to many children like me… Did I die? No, I am still alive. Why should you frequent the hospital?’”* (Female FGD participant, Maper centre)
*"Some women do not go to the hospital because their husbands do not allow them to.”* (Female FGD participant, Meen village)
    Moreover, women felt that they were not getting the encouragement and support they needed during pregnancy and that men lacked interest in maternal health; leaving the burden of taking care of the pregnancy to women.
*“Here if you are pregnant, men don’t care. They just leave the pregnancy for you alone. They leave you for the rest of the pregnancy*.” (Female FGD participant, Madhol village)
    Some men encouraged their wives to attend ANC. However, this was primarily driven by the need to have their wives get tested for any disease that would affect the foetus or just to check the status of the foetus. There was little concern about the health of the mother.
*“Visiting the clinic during pregnancy is useful because it helps the pregnant woman and her husband to know the gender of the baby, the time of delivery and to determine whether she is carrying twins.”* (Male FGD participant, Meen village)
*“Attending the hospital is for checking the foetus inside the woman’s womb; that is to find out if the foetus is free from diseases or not. If the foetus has a disease, the hospital provides treatment.”* (Male FGD participant, Ror bar village)
  Insecurity
    Rumbek North is in a state of chronic insecurity and the inhabitants live in fear of being attacked at any time by the neighbouring communities. Although the county is known for frequent inter-clan feuds, the prevailing political and security situation in South Sudan had exacerbated the problem. Insecurity was widely cited as the reason behind poor ANC utilisation.
*“The fighting is really affecting us; for example, now we have heard that people from the neighbouring clan have attacked Madhol village. Do you think that if a pregnant woman had planned to come today she will come? She will not come because of fighting. Many people are attacking our village every day and for that reason, we cannot go to a distant hospital….”* (Female FGD participant, Maper centre)
C. Perceptions of pregnancy
  Perceived benefit
    ANC was perceived in the county to be a new concept. The population had lived for a long time without formal health care services and some women had never visited a health facility. Consequently, some women were not familiar with ANC and its significance.
*“The reason why others don’t go to the hospital is that the doctors have just come recently. Our villages have been in the forest all along. For example, Maper never had a hospital in the past. So many people don’t know the goodness of the hospital and that is why they do not use it.”* (Female FGD participant, Chatom village)
*“I have never gone to the hospital ever since I was born and so I don’t know much about the hospital.”* (Female FGD participant, Madhol village)
    Additionally, in the case of problems during pregnancy, some women turned to traditional remedies because they did not know about the medical treatment in health facilities.
*“I don’t know the importance of the hospital. During my previous pregnancy, whenever I had any kind of illness, I went to the traditional healer.”* (Female FGD participant, Chatom village)
    The health facility staff felt that efforts to raise awareness, though still limited, were resulting in an increase in the number of women attending ANC.
*“Last year they were not attending but this year they are. This is because previously, they were unaware of the benefits of the health facility. We have tried to create awareness in the villages and they are listening and starting to understand. A few women came, received good care and went back to inform their colleagues to come.”* (KII, CHW, Maper PHCC)
  Perceived risk
    The risk of serious pregnancy-related complications was perceived to be low. Some women without any prior pregnancy related complication did not see the added value of ANC attendance in the context of the formidable barriers to service access. Similarly, those with a health concern did not perceive the problem to be a major threat to the pregnancy.
*“Some of us who did not experience any problem during the first pregnancy don’t see the need of going to the hospital. We reason that the present pregnancy will be like the first one.”* (Female FGD participant, Ror bar village)
    On the other hand, women with prior pregnancy-related complications were more likely to attend ANC because of their higher risk perception.
*“For my first pregnancy, my child died because of the sickness I was having during pregnancy. From there, I said to myself that for my second pregnancy, I would not want my baby to die again. I went to the hospital for check-up and treatment, came back home and delivered safely to a life child.”* (Female FGD participant, Wundhiot village)
    Attending ANC was often associated with medical treatment. As seen above, FGD participants seldom viewed ANC as a preventive service. On one hand this promoted ANC attendance for women with health problems but on the other hand, it was a potential reason to avoid service use for women who did not feel any discomfort.
*“I visited the clinic when I was pregnant because I was very sick. While there, I was injected with medicine and then I felt well.”* (Female FGD participant, Maper centre)
    Some male participants had a positive perception toward ANC especially the medical care received at health facilities—which they felt sometimes benefited them too.
*“We think that it is good for a pregnant woman to visit the health facility because sometimes she may be having fever and body pain and this will require her and the husband to visit a health facility for treatment. Accompanying a woman may encourage her to attend the clinic. Both of you will be tested together. You may be suffering from a sexually transmitted disease and this will require the doctor to treat both of you.”* (Male FGD participant, Maper centre)
D. Perceived low quality of care and poor efficacy of medical treatment
  Because ANC was perceived be a curative service, some women felt unsatisfied if treatment was not provided during ANC while others were unsatisfied with the quality of treatment provided if it did not include an injection or if the treatment did not relieve the symptoms experienced. These issues seemed to affect decisions on future ANC attendance.
*“What we want is medicine. Even if you go to the hospital, you will not be given medicine. I have gone to the hospital twice but nothing has happened. Now I have decided to go to Marial lou hospital because this medicine of Maper hospital is not helping.”* (Female FGD participant, Nhomleng cattle camp)
*“Some pregnant women prefer to be injected. If you give them a tablet, they don’t accept because they think that only injectable drugs can help them.”* (KII, CHW, Meen PHCU)

## Discussion

In general, the barriers to utilisation of ANC services identified in the present study have also been reported in other studies [16]. Some of them, such as transportation problems and insecurity, affect both service provision and utilisation; aggravating the problem of poor access to maternal health services. In this setting, geographical barriers and insecurity made it difficult and dangerous for women to travel to health facilities thereby negatively influencing the decision to attend ANC.

Although maternal health services were officially free-of-charge in Rumbek North, women were discouraged from utilising ANC services because health workers at some health facilities were demanding for payment. One study in South Sudan has reported that “under-the-table” payments were hindering institutional childbirth in the country [17]. Any out-of-pocket payment requested, either official or illegal, has a considerable negative impact and dramatically hinders the delivery of ANC in this setting. An investigation into the reasons behind these possible “under-the-table” payments is warranted.

The present study identified two key sociocultural barriers to ANC utilisation: women’s domestic chores and the influence of male partners/husbands. Both of these issues are reflective of the women's empowerment situation in this community. Women were overwhelmed with domestic chores and had little or no time to attend ANC. Surprisingly, the issue of women’s domestic chores did not emerge in men’s FGDs. Husbands were perceived to be unsupportive or restrictive of their wives attending ANC. Although some men were supportive of ANC attendance, this had a lot to do with the health of the foetus rather than that of the mother. We have previously reported that husbands were restricting their wives from utilising childbirth services in this setting [10]. This restriction also applied to ANC, although to a lesser degree.

Tradition and culture had a formidable influence on institutional childbirth in this setting [10] but seemed to have a relatively weaker influence on ANC attendance. This is probably because ANC was perceived to be important in confirming the wellness of the baby, and for the treatment of health problems. The relationship between the health of the woman and the health of the baby seemed to be poorly understood. Pregnancy was perceived to be a normal life event that did not require visiting a health worker unless if there was a complication. Thus most participants related ANC to the treatment of illnesses during pregnancy as has been documented elsewhere [16]. This perception that ANC was a curative rather than a preventive health service might result into sporadic utilisation driven by the perceived severity of health problems during pregnancy. This same perception determined the criteria women applied to judge the quality of care received. Because most women attended ANC at PHCUs and these health facilities stocked only non-injectable drugs in line with the government guidelines, the medical treatment received was perceived to be inadequate and a waste of time. As has been reported elsewhere [16, 18], perceptions of medical interventions during previous ANC shaped the attitude towards the future use of ANC.

Lack of correct and sufficient information about the importance of maternal health care and poor understanding of the risks aggravates the socio-cultural barriers mentioned above. Most women in Rumbek North, especially those in cattle camps, had no prior experience of ANC and were unsure about its utility. The community was also generally poorly informed about maternal health care. Campaigns to raise awareness not only among women but also in the entire community may be essential to overcome, in a reasonable timeframe, the effects of the lack of knowledge and improve the understanding of the crucial role of ANC as has been demonstrated elsewhere [19, 20].

In low-resource settings, pregnancy is often one of the rare occasions on which women establish contact with the health system and thus it is a unique opportunity to deliver evidence-based care and interventions that could be lifesaving [21]. In 2015, the maternal mortality ratio in South Sudan was estimated by WHO to be 789 maternal deaths per 100,000 live births [22]. With this very high maternal mortality, it is crucial to identify effective and sustainable strategies to reach women and improve the delivery of care practices during pregnancy and childbirth in this country.

This is the first study to document, in detail, the barriers to ANC use in Rumbek North and one of the few in-depth inquiries into barriers to ANC service access and use in South Sudan. This is an important contribution to understanding the reasons for poor maternal health service use in the country. To ensure effectiveness, the development of any strategies to improve access to ANC needs to take into account the barriers clearly identified by this study. We used two complementary methods to collect data from diverse sources. The diversity of the respondents and the triangulation of the data ensured that the main perceptions prevalent in the community were captured. We believe the findings of this study would apply to other counties in South Sudan with similar characteristics as Rumbek North. Some of the findings may also apply to conflict-affected settings. This study, however, has some limitations. First, it is possible that some information was lost in translation and transcription. Nevertheless, this problem might have been mitigated through triangulation of data from different sources. Secondly, we limited our study to examining barriers to utilisation of any antenatal care. A further inquiry could look at barriers to late booking and to attending the recommended number of ANC visits.

## Conclusions

Multiple factors and their interactions affect access to and utilisation of ANC services in Rumbek North County in South Sudan. Some of the factors, such as insecurity and poor geographical accessibility, are outside the scope of the health sector and require a multi-sectoral approach. Restoration of peace in South Sudan will also require the support of the international community. The health system in Rumbek North, as in other parts of the country, is still evolving, and health services planners should consider how to address the barriers identified in this study in their strategies to improve ANC service utilisation. Creating demand for ANC service will need to be done alongside investment in the supply side, especially in human resources and in health infrastructure, to meet the potential future increase in demand. The findings of this study will inform CUAMM’s strategy as the organisation works with the Rumbek North CHD and other stakeholders in ensuring that ANC services are available and accessible to the residents of the county. More studies on this subject in South Sudan are still needed to provide a comprehensive understanding of barriers to ANC to better inform strategies to address the problem.

## Additional file

Additional file 1: Roles of women and men according to source of information. (DOCX 15 kb)

## Abbreviations

ANC: Antenatal care
BPHNS: Basic Package of Health and Nutrition Services
CHD: County Health Department
CHW: Community health worker
COREQ: Consolidated criteria for reporting qualitative research
CUAMM: Collegio Universitario Aspiranti e Medici Missionari
FGD: Focus group discussion
HIV: Human immunodeficiency virus
HSDP: Health Sector Development Plan
KII: Key informant interview
MoH: Ministry of Health
PHCC: Primary health care centre
PHCU: Primary health care unit
SHHS: Sudan Household Survey
TBA: Traditional birth attendant
WHO: World Health Organization
