# Supplementary material for: Barriers to utilisation of antenatal care services in South Sudan: a qualitative study in Rumbek North County
Source: Reprod Health. 2017 May 22;14:65. doi: 10.1186/s12978-017-0327-0 (PMC5440928; doi:10.1186/s12978-017-0327-0)
Supplement: Additional file 1: — Roles of women and men according to source of information. (DOCX 15 kb) [file 12978_2017_327_MOESM1_ESM.docx]

Additional File 1: Roles of women and men according to source of information

| **Roles of women** | | | **Roles of men** | | |
| --- | --- | --- | --- | --- | --- |
| **Role** | **Female FGDs** | **Male FDGs** | **Role** | **Female FGDs** | **Male FDGs** |
| Cooking food | X | X | Clearing the land for cultivation | X | X |
| Cleaning the house/compound | X | X | Taking care of cattle at home and in cattle camp | X | X |
| Building the house | X | X | Cutting big trees | X |  |
| Thatching the house | X | X | Enforcing family rules | X |  |
| Clearing the land | X |  | Making a woman pregnant | X |  |
| Cultivation of land | X | X | Setting up a new compound | x |  |
| Planting | X |  | Community/family security | X | X |
| Weeding crops | X |  | Cultivating using the ox | X |  |
| Harvesting crops | X |  | Head of the family | X | X |
| Cutting grass for thatching | X |  | Looking for lost cows | X | X |
| Taking care of children | X | X | Laying bricks | X |  |
| Taking care of visitors | x | X | Selling cattle to meet the family needs | X | X |
| Taking care of the husband | X | X | Building the house and the fence |  | X |
| Taking care of animals at home | X |  | Looking for cattle to pay dowry |  | X |
| Collecting firewood | X |  |  |  |  |
| Milking | X |  |  |  |  |
| Fetching water | X | X |  |  |  |
| Processing food (Pounding grains, drying groundnuts etc.) | X | X |  |  |  |
| Collecting wild fruits | X |  |  |  |  |
| Washing clothes | X |  |  |  |  |
| Childbirth |  | x |  |  |  |
| Preparing beddings |  | X |  |  |  |
| Advising the man |  | X |  |  |  |
| Store keeper |  | X |  |  |  |

X means the role was mentioned and a blank space means the role was not mentioned.
